# Clinical, cytogenetic, and genomic analyses of an Ecuadorian subject with Klinefelter syndrome, recessive hemophilia A, and 1;19 chromosomal translocation: a case report

**DOI:** 10.1186/s13039-022-00618-w

**Published:** 2022-09-05

**Authors:** Anibal Gaviria, Santiago Cadena-Ullauri, Francisco Cevallos, Patricia Guevara-Ramirez, Viviana Ruiz-Pozo, Rafael Tamayo-Trujillo, Elius Paz-Cruz, Ana Karina Zambrano

**Affiliations:** 1 Centro de Investigación Genética y Genómica, Facultad de Ciencias de la Salud Eugenio Espejo, Universidad UTE, Quito, Ecuador; 2 Laboratorio de Genética Molecular, Centros Médicos Especializados Cruz Roja Ecuatoriana, Quito, Ecuador; 3 Hemocentro Nacional, Cruz Roja Ecuatoriana, Quito, Ecuador

**Keywords:** Klinefelter syndrome, Hemophilia A, Chromosomal translocation

## Abstract

**Background:**

Hemophilia A is considered one of the most common severe hereditary disorders. It is an X-linked recessive disease caused by a deficiency or lack of function of the blood clotting factor VIII. Klinefelter syndrome is a genetic disorder that affects male individuals due to one or more extra X chromosomes, present in all cells or with mosaicism. The aneuploidy is due to either mitotic or meiotic chromosome non-disjunction. Chromosomal translocations are a group of genome abnormalities in which a region or regions of a chromosome break and are transferred to a nonhomologous chromosome or a new location in the same chromosome.

**Case presentation:**

Our subject was born in Ecuador at 36 weeks of gestation by vaginal delivery. At 3 months old, the Factor VIII activity measure showed a 23.7% activity indicating a diagnosis of mild hemophilia A. At 1 year old, the karyotype showed an extra X chromosome, consistent with a diagnosis of Klinefelter syndrome, and a translocation between the long arms of chromosomes 1 and 19, at positions q25 and q13, respectively.

**Conclusions:**

Klinefelter syndrome and hemophilia are a rare combination. In the present case report, the subject presents both, meaning that he has inherited one X chromosome from the father and one X chromosome from the mother. Since the father has severe hemophilia A; and the subject presents a below 40% Factor VIII activity, a skewed X inactivation is suggested. Additionally, the proband presents a translocation with the karyotype 47,XXY,t(1;19)(q25;q13). No similar report with phenotypic consequences of the translocation was found. The present report highlights the importance of a correct diagnosis, based not only on the clinical manifestations of a disease but also on its genetic aspects, identifying the value of integrated diagnostics. The subject presents three different genetic alterations, Klinefelter syndrome, hemophilia A, and a 1;19 chromosomal translocation.

**Supplementary Information:**

The online version contains supplementary material available at 10.1186/s13039-022-00618-w.

## Background

Hemophilia A is considered one of the most common severe hereditary disorders. One of the main characteristics of this disorder is prolonged bleeding after minor injuries; in some cases, the bleeding could be spontaneous. The reports in Ecuador indicate a Hemophilia A frequency of 1:5000 to 1:10,000 cases per male birth [1].

Hemophilia A is an X-linked recessive disorder caused by a deficiency or lack of function of the blood clotting factor VIII due to a mutation in the F8 gene located in the long arm of chromosome X [2, 3]. Generally, female carriers will transmit the defective gene to half of the male offspring, in which case they will present the disorder; whereas, in contrast, male hemophilia A patients do not pass on the defective gene to the male offspring, and the female offspring will be carriers [4]. However, in some cases, male hemophilia A patients could pass on the disorder if they transmit the defective X chromosome.

During the coagulation process, the role of factor VIII is to enhance fibrin formation and thrombin generation to prevent further bleeding [4]; the severity of the disorder depends on the level of activity of the factor VIII [5].

Klinefelter syndrome is a genetic disorder that affects male individuals due to one or more extra X chromosomes present in all cells or with mosaicism. The aneuploidy is due to either mitotic or meiotic chromosome non-disjunction [6, 7]. However, due to X inactivation, only one X chromosome will be completely functional [8]. The estimated prevalence of Klinefelter syndrome in Ecuador is 1:500 to 1:1000 in males [9, 10].The clinical features of the patients include a broad spectrum of manifestations involving a short trunk, firm testes, gynecomastia, hypogonadism, testosterone deficiency, disorders of testicular descent, and infertility [6, 7, 11]. Moreover, the patients have an increased mortality and morbidity risk associated with propensity to embolism, increased prevalence of diabetes, osteoporosis, autoimmune diseases, bone fractures, and specific types of cancer [7]. Cognitive delays such as deficits in attention and verbal processing have also been associated with Klinefelter syndrome [11].

Chromosomal translocations are a group of genome abnormalities in which a region or regions of a chromosome break and are transferred to a nonhomologous chromosome or a new location in the same chromosome [12, 13]. The consequences of the translocations depend on the location of the break; these include gene dysregulation, direct gene disruption, and the creation of a fusion gene. Furthermore, at the phenotypic level, translocations could lead to infertility, aneuploidy, or cancer [14]. Karyotyping is used for the detection of translocations and changes in chromosome number [15].

This report describes the clinical manifestations, cytogenetic, and genomic analyses of a subject diagnosed with Klinefelter syndrome, hemophilia A, and a translocation between chromosomes 1 and 19. The objective is to improve the understanding of the molecular biology, physiology, and interaction of these disorders.

## Case presentation

**Fig. 1:**
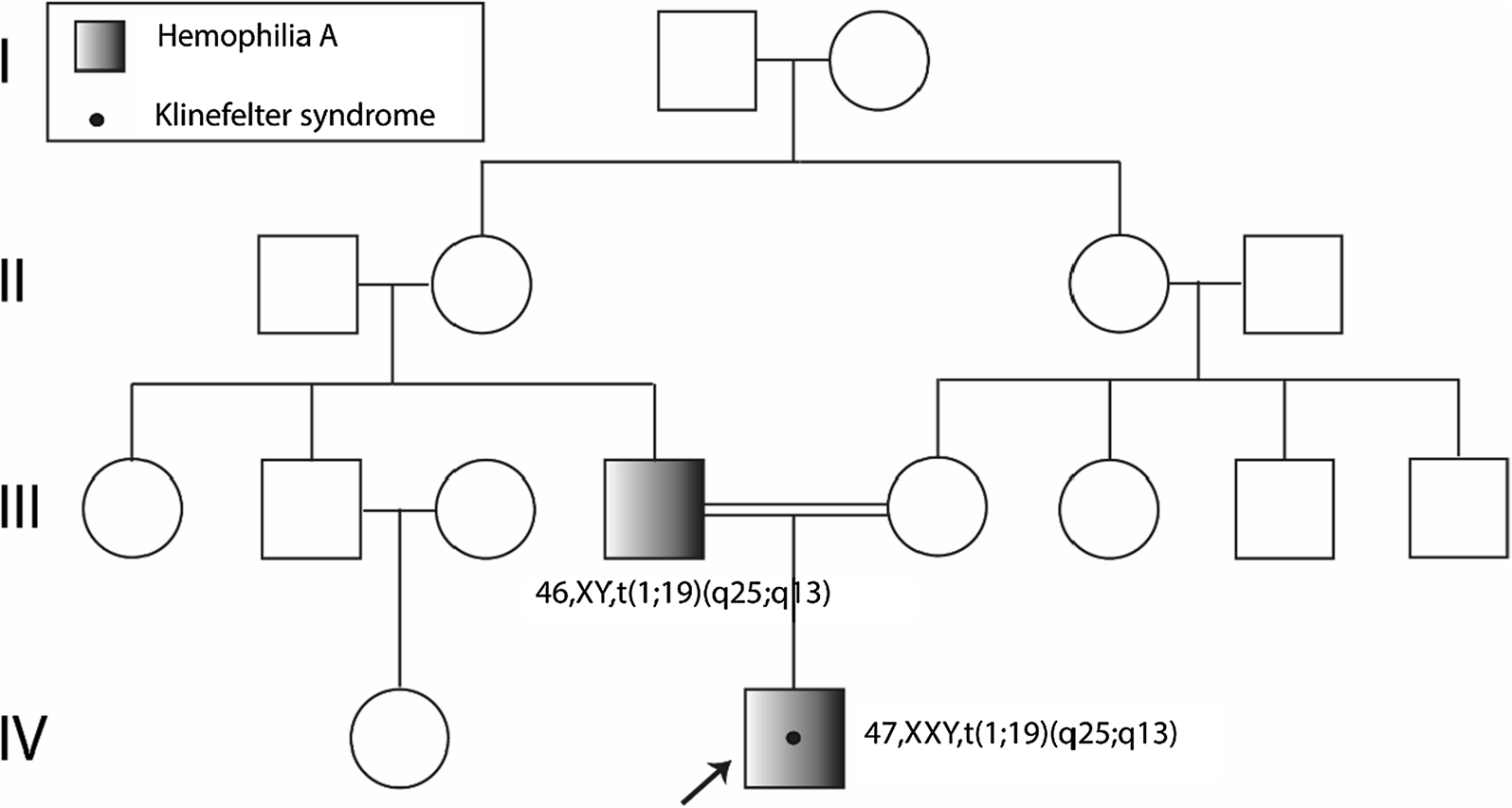
Proband pedigree. The proband, indicated with an arrow, has a diagnosis of Hemophilia A, Klinefelter syndrome, and t(1;19)(q25;q13). Similarly, the proband’s father has a diagnosis of Hemophilia A and carries the same translocation

The subject was born in Guayaquil, Ecuador, at 36 weeks of gestation by vaginal delivery. His weight was 2.5 kg, length was 46 cm, and head circumference was 35.5 cm (normal values for gestational age). The mother did not report complications during the pregnancy; she was 23 years old at the subject’s birth. The father has been diagnosed with severe hemophilia A, and no other family members reported to be affected by any other disease (Fig. 1).

At the time of birth, the subject presented a hematoma in the forehead, which, based on the father’s diagnosis of hemophilia, raised doubts about the child status. At 3 months old, the Factor VIII activity measure showed a 23.7% activity indicating a diagnosis of mild hemophilia A. Moreover, a Factor V activity test was performed, showing normal ranges of activity, rejecting a diagnosis of hemophilia B. At 5 years old, the Factor VIII activity was measured again, showing a 38.8% activity and 0.00 Bethesda Units/mL of Factor VIII inhibitor. The treatment has consisted of Factor VIII ampoules, administered only in the presence of hematomas.

**Fig. 2:**
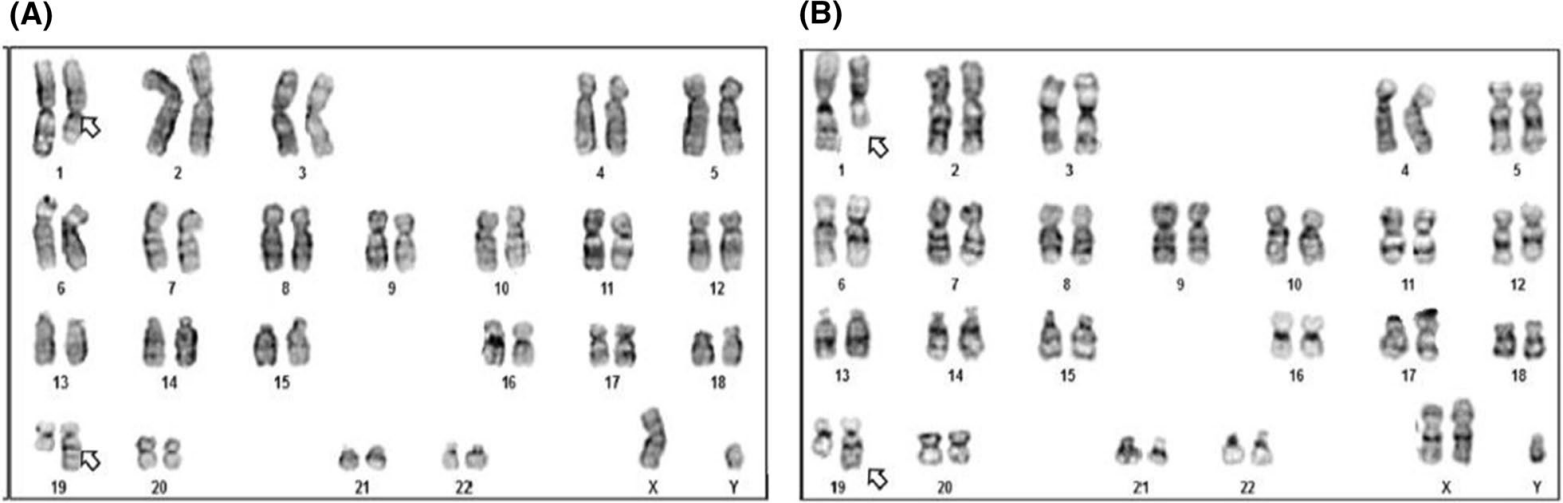
Proband and proband’s father karyotype. **A** Proband’s father karyotype shows the translocation between chromosomes 1 and 19, indicated with arrows (46,XY,t(1;19)(q25;q13)). **B** Proband karyotype shows an extra X chromosome and a translocation between chromosomes 1 and 19 indicated with arrows (47,XXY,t(1;19)(q25;q13))

The karyotype from lymphocyte cultures, at 450-band resolution, when the patient was 1 year old revealed a translocation between the long arms of chromosomes 1 and 19, at positions q25 and q13 (t(1;19)), and Klinefelter Syndrome, as follows: 47,XXY,t(1;19)(q25;q13), (illustrated in Fig. 2). The mother showed a normal karyotype while the father exhibited the same translocation as the proband, depicted in Fig. 2.

Moreover, an X chromosomal Short Tandem Repeat (STR) markers study was carried out on the three individuals (mother, father, and proband). DNA extraction was performed from peripheral blood samples with PureLink Genomic DNA mini kit, according to the manufacturer’s recommendations (Invitrogen™). Investigator Argus X-12 QS and X-STR Decaplex were carried out according to the manufacturer’s instructions, and as reported by a GEP-ISFG collaborative study [16], respectively. Capillary electrophoresis, in an Applied Biosystems 3500 Genetic Analyzer 8-Capillary Array (Thermo Fisher Scientific), was used for amplicon separation. The data were collected with DataCollection v. 3.3 and analyzed with GeneMapper v. 5 (Applied Biosystems) The proband’s results presented heterozygosity in different X-STRs, confirming the double dose of the X chromosome, where one X chromosome came from the mother and one X chromosome from the father. For example, in the marker DXS10103, the mother is homozygous for the allele 16, the father is homozygous for the allele 18, the proband has both alleles 16 and 18; hence, the allele 18 was inherited from the father (Additional file 1: Table S1).

At 5 years old, follicle-stimulating hormone (FSH) and Luteinizing hormone (LH) levels were measured. The FSH levels were 1.06 mIU/mL (normal). LH levels were 1.00 mIU/mL, showing higher than normal values, supporting the diagnosis of KS. The subject underwent Spanish language development tests; the results evaluating phonological simplification processes (TEPROSIF) and the screening test of Spanish grammar (STSG) receptive and expressive showed a deficit, indicating results of someone within the 2 years old range. The diagnosis was a mixed specific language impairment, in accordance with the KS diagnosis. No treatment for the condition has started yet, given that it is recommended to start at early puberty.

To further understand the extent of the possible consequences of the chromosome 1 and 19 translocation, including insertions, deletions, and single-point mutations, Next-Generation sequencing (NGS) analyses were performed using the subject peripheral blood sample. The TruSight Inherited Disease panel (Illumina) was used; which covers 552 genes associated with severe, pediatric-onset diseases [17]. The results showed 1 341 variants that passed the quality filters; however, none of these variants were reported to be pathogenic or related to the subject’s symptomatology. No significant loss of genomic content was found, in LAMC2, and NPHS2 genes located in the q25 region of chromosome 1, neither in HAMP, COX6B1, NPHS1, DLL3, PRX, BCKDHA, ETHE1, ERCC2, OPA3, FKRP, NUP62, ETFB genes located in the q13 region of chromosome 19 (data not shown).

## Discussion

Klinefelter syndrome and hemophilia are a rare combination. The symptoms vary and based only on the phenotypic characteristics, a diagnosis could be difficult, especially during the first years of life [6, 7, 11]. Early diagnosis has been correlated with a decrease in the morbidity and mortality of Klinefelter syndrome [11].

In the present case report, a karyotyping test confirmed the diagnosis of KS, showing the presence of an extra X chromosome. Furthermore, the subject exhibits symptoms associated with KS like hypogonadism, disorder of testicular descent, and language impairment. Hemophilia A is an X-linked recessive disorder; therefore, males are the most affected, since they have only one X chromosome, inherited from the mother. However, in the present case report, the subject presents hemophilia A and KS. He has inherited one X chromosome from the father and one X chromosome from the mother, based on the X chromosomal Short Tandem Repeat (STR) markers study (Additional file 1: Table S1), where paternal and maternal markers were found in the proband. In females carrying two X chromosomes, during early fetal life, one of the chromosomes will be randomly inactivated. On average, 50% of the paternal chromosome and 50% of the maternal chromosome will be inactivated in all the somatic cells [18]. Similarly, this process occurs in individuals with KS, where one of the chromosomes X will be transcriptionally inactive [18, 19]. Since the father has severe hemophilia A; and the subject presents a below 40% Factor VIII activity, a skewed X inactivation is suggested; where the cells are inactivated in mosaicism. The mutation causes a factor VIII deficiency in plasma; the degree of deficiency corresponds to the bleeding symptoms. The subject presents mild hemophilia based on the activity percentage of Factor VIII (38.8%); in this case bleeding starts after a significant injury or surgery [4, 5].

Additionally, the proband presents a translocation with the karyotype (47,XXY, t(1;19)(q25;q13)). No similar reports with phenotypic consequences of the translocation were found. A correlation between the father and son symptoms was analyzed; however, no symptoms could be attributed to the translocation, considering the father presents the same translocation but only shows hemophilia A manifestations. NGS analyses were performed to further understand the consequences of the translocation. However, no significant variants were found, and no genomic content loss was identified. There are no reports of the same translocation; although, translocations involving the same chromosomes have been correlated with a higher risk of developing cancer and infertility [15]. The consequences of the translocation are not fully understood. Hence, it is of the utmost importance to maintain a careful vigilance of the signs and symptoms the subject may present.

The present report highlights the importance of a correct diagnosis, based not only on the clinical manifestations of a disease but also on its genetic aspects, identifying the value of integrated diagnostics. The subject presents three different genetic alterations, Klinefelter syndrome, hemophilia A, and a chromosome 1–19 translocation. These disorders have been correlated with distinct symptoms, including additive negative consequences in the subject; hence, it is vital to undergo routine checkups to determine the best course of action and to intervene on time with the correct treatment.

## Supplementary Information

**Additional file 1**. Table 1. X-chromosome markers and amelogenin.

## Data Availability

The results are presented in the paper. For more information, please contact the corresponding author A.K.Z. (anazambrano17@hotmail.com).
