# Supplementary material for: Clinical, cytogenetic, and genomic analyses of an Ecuadorian subject with Klinefelter syndrome, recessive hemophilia A, and 1;19 chromosomal translocation: a case report
Source: Mol Cytogenet. 2022 Sep 5;15:40. doi: 10.1186/s13039-022-00618-w (PMC9446752; doi:10.1186/s13039-022-00618-w)
Supplement: Supplementary file 1 — Additional file 1. Table 1. X-chromosome markers and amelogenin. [file 13039_2022_618_MOESM1_ESM.docx]

**Table 1. X-chromosome markers and amelogenin**

| **STR** | **Mother** | **Son** | **Father** |
| --- | --- | --- | --- |
| **DXS10103** | 16-16 | 16-**18** | 18 |
| **DXS8378** | 10-11 | 10-10 | 10 |
| **DXS10101** | 32-33 | **30.2**-33 | 30.2 |
| **DXS10134** | 35-37 | **36**-37 | 36 |
| **DXS10074** | 8-15 | 15-**16** | 16 |
| **DXS7132** | 13-15 | **14**-15 | 14 |
| **DXS10135** | 11-25 | 25-**33** | 33 |
| **DXS7423** | 15-15 | 15-15 | 15 |
| **DXS10146** | 28-28 | **25**-28 | 25 |
| **DXS10079** | 17-20 | **14**-20 | 14 |
| **HPRTB** | 12-13 | 12-13 | 13 |
| **DXS10148** | 18-26 | 18-26 | 18 |
| **DXS9898** | 12-12 | 12-12 | 12 |
| **DXS7133** | 9-9 | 9-9 | 9 |
| **GATA31E08** | 12-12 | 12-12 | 12 |
| **GATA172D05** | 11-11 | 11-11 | 11 |
| **DXS6809** | 33-34 | 33-34 | 34 |
| **DXS9902** | 11-12 | 11-12 | 12 |
| **DXS6789** | 16-20 | 20-20 | 20 |
| **AMELOGENIN** | X-X | X-Y | X-Y |

* The alleles that could be determined to be inherited from the father are in Bold.
